# Contact Behavior of Composite CrTiSiN Coated Dies in Compressing of Mg Alloy Sheets under High Pressure

**DOI:** 10.3390/ma11010088

**Published:** 2018-01-08

**Authors:** T.S. Yang, S.H. Yao, Y.Y. Chang, J.H. Deng

**Affiliations:** 1 Department of Mechanical and Computer Aided Engineering, National Formosa University, Yunlin 632, Taiwan; tsyang@nfu.edu.tw (T.S.Y.); yinyu@nfu.edu.tw (Y.Y.C.); wuuboss123@gmail.com (J.H.D.); 2 Chang Jung Christian University, Tainan 71101, Taiwan

**Keywords:** CrTiSiN coating, Mg alloy, cathodic arc deposition process, wear test, compression, friction test

## Abstract

Hard coatings have been adopted in cutting and forming applications for nearly two decades. The major purpose of using hard coatings is to reduce the friction coefficient between contact surfaces, to increase strength, toughness and anti-wear performance of working tools and molds, and then to obtain a smooth work surface and an increase in service life of tools and molds. In this report, we deposited a composite CrTiSiN hard coating, and a traditional single-layered TiAlN coating as a reference. Then, the coatings were comparatively studied by a series of tests. A field emission SEM was used to characterize the microstructure. Hardness was measured using a nano-indentation tester. Adhesion of coatings was evaluated using a Rockwell C hardness indentation tester. A pin-on-disk wear tester with WC balls as sliding counterparts was used to determine the wear properties. A self-designed compression and friction tester, by combining a Universal Testing Machine and a wear tester, was used to evaluate the contact behavior of composite CrTiSiN coated dies in compressing of Mg alloy sheets under high pressure. The results indicated that the hardness of composite CrTiSiN coating was lower than that of the TiAlN coating. However, the CrTiSiN coating showed better anti-wear performance. The CrTiSiN coated dies achieved smooth surfaces on the Mg alloy sheet in the compressing test and lower friction coefficient in the friction test, as compared with the TiAlN coating.

## 1. Introduction

Mg alloys feature low density (pure Mg ~1.738 g/cm^3^), high strength/weight ratio, good machinability, anti-shock performance and corrosion resistance and easy recycling. They have been used in various fields from aerospace plane parts to daily use products. However, Mg alloys are hard-to-deform metals due to poor plastic deforming ability resulting from hexagonal close packed (HCP) structure, which makes short tools service life. Surface hard coating is one of the ways to increase the tool life. Industrial applications of hard coatings in a form of either single layer or multilayer synthesized by physical vapor deposition (PVD) have been increasing rapidly because they can be prepared relatively easily and provide improvements in tribological or corrosive properties [1,2], or high temperature oxidation resistance [3]. Among diversely developed PVD techniques, the cathodic arc deposition technique features coatings with good adhesion and high deposition rate due to their high ionization and high current density as compared with other deposition processes [4]. However, it should be addressed that the properties of the obtained coatings strictly depend on the technological parameters applied in the deposition processes. Under some cases, the coatings obtained by using other PVD methods may show better performance than the ones by cathodic arc deposition technique [5,6].

Chromium nitride (CrN) coatings have been applied in molding and machining industries to prolong tool service life because they show excellent adhesion [7], corrosion resistance, and anti-wear and machining performance [8,9]. CrN coatings have been noted for its good oxidation resistance up to 800 °C [10]. Thus, CrN coatings are good candidates for hard forming metals such as Mg or Ti alloy or some specific alloys like Monel, Inconels, or Hastelloy [8]. However, it has been learned their hardness is slightly lower than that of other hard coatings [9,11]. The concept of nanocomposite hard coating Cr-X-N has been used for hardness improvement, where X is an addition such as Ti, Al, Si, B, C, Ta, Nb, or Ni [8]. The addition of Si to the CrN coating to form amorphous Si_3_N_4_ phase has been adopted to improve the mechanical properties and oxidation resistance [12,13,14,15]. It has been observed that CrN crystalline and amorphous Si_3_N_4_ phase are two major contributors in the CrSiN nanocomposite coating [13,15]. A study on CrSiN coatings indicated their microhardness and micro wear resistance can be enhanced with Si addition of up to 12% [15]. For the Ti addition, the mechanical properties of binary metal nitride coatings can be enhanced due to solid solution hardening effects [16]. With the advance in PVD technology, the design and application are in full swing of ternary, quaternary and multicomponent metal nitride nanostructure and nanocomposite coatings.

TiN base coatings have been widely used in the tool industries as protective coatings because they provide increased wear resistance and cutting accuracy [17,18]. Numerous kind of TiN base coatings have been developed with variations in composition and structure. When encountering severe conditions—high loading friction or speed machining, the first candidate is TiAlN coatings because they show high oxidation resistance at elevated temperatures combined with good wear resistance due to the formation of Al_2_O_3_ layer [19,20]. In practice, single layer TiAlN coatings are still the most generally used. The characteristic features of TiAlN coatings could be controlled by adjusting chemical composition and microstructure that significantly influence their mechanical properties [21]. In literature, there have been many documents dealing with analyses and applications of TiAlN coatings [22,23,24,25].

In the existing literature, very few papers discussed the CrTiSiN coatings. The only one that the authors found was by Ho et al. [26] but on its wear and corrosion properties. In this study, a cathodic arc deposition process was used to prepare a composite CrTiSiN hard coating. One Ti/Si (50/50%) target was used to achieve the addition of third and fourth elements into CrN coating. A TiAlN coating was prepared as well for comparison. Microstructure, adhesion and wear performance were studied. A compression and friction testing system was designed and assembled to evaluate the contact behavior of CrTiSiN coated dies in compressing of the Mg alloy sheets. The surface conditions of Mg sheets after compression and friction behavior during compressing were studied. 

## 2. Experiment

### 2.1. Coating and Characterization

A twin-cathodic arc evaporation system was used to prepare the coatings. The Ar and H_2_ gas were introduced through a conducting duct around the targets to enhance the reaction of the plasma and to reduce the droplets within the deposited coatings. Two targets (cathodes), Cr (99.9%) and Ti/Si (50/50%), were used and arranged on the opposite sides of deposition chamber. The distance from the cathodes to substrates was 180 mm. When the deposition system was on, the substrate holder speed was kept constant at 4 rpm. In the beginning, the base pressure was vacuumed to lower than 2 × 10^−5^ mbar. Next, the Ar gas was introduced to a pressure 10^−2^ mbar, and then the H_2_ gas was introduced and arc was triggered at the same time to obtain Ar^+^ and H^+^ ions. The chamber temperature was mainly controlled by the H^+^ ion density. The H_2_ gas was kept introduced to elevate the chamber average temperature reaching 300 °C and keeping fixed. The temperature was measured by a thermocouple located near the samples. Prior to deposition, the samples were sputter cleaned using the Ar^+^ and H^+^ ions under bias voltage V_b_ −1000 V for 30 min. Subsequently, the coating started to grow. The Cr interlayer was prepared first under conditions of Cr target current 60 A, substrate bias voltage V_b_ −120 V and deposition duration 10 min. Then, the gradient CrN layer was prepared with N_2_ introduction at pressure 0.027 mbar for 10 min while the other conditions were left unaltered. Finally, the outmost CrTiSiN layer was grown by applying current 60 A to both the Cr and Ti/Si targets for 40 min while kept the other conditions unaltered. The referenced traditional TiAlN hard coating was deposited using the same deposition system. Two targets, Ti (99.9%) and Ti/Al (50/50%), were used and arranged on the opposite sides of deposition chamber. By using the same conditions as for the CrTiSiN coating, the Ti interlayer, TiN layer and outmost TiAlN layer were prepared. The conditions for preparing the Ti interlayer, TiN layer and outmost TiAlN layer (50 min) were the same as for the Cr interface, CrN layer and outmost CrTiSiN layer, respectively. 

**Figure 1:**
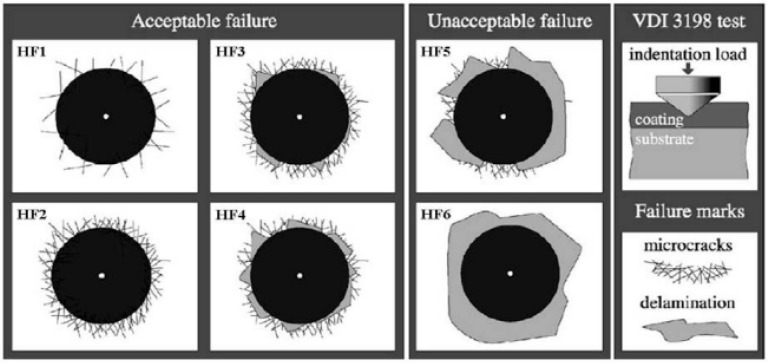
VDI 3198 norm, used to make destructive quality test by means of Rockwell C hardness indentation tester (Reproduced with permission of the Verein Deutscher Ingenieure e. V.) [28].

A Jeol JSM-7000F high resolution field emission scanning electron microscope (FESEM) (Jeol, Chiyoda, Tokyo, Japan) was used equipped with an energy-dispersive X-ray spectroscopy (EDS). The chemical composition was analyzed using EDS. The hardness (*H*) and Young’s modulus (*E*) of each indent were obtained using a Nano-indenter test (TI 950 TriboIndenter) with a Berkovich indenter on the basis of the Oliver and Pharr method [27]. The conditions were used of maximum applied load 50 mN at a loading-unloading rate of 20 mN/s. The Young’s modulus, *E*, was expressed as follows: 1/*Er* = (1 − *v*^2^)/*E* + (1 − *v_i_*^2^)/*E_i_*, where *Er* and *v* were the reduced Young’s modulus and assumed Poisson ratio 0.25 for the coatings evaluated, and *E_i_* (1140 GPa) and *v_i_* (0.07) were the corresponding parameters of the diamond indenter. The well-known Rockwell C hardness indentation test was employed to evaluate the adhesion of coatings. It is a destructive quality test by penetrating a conical diamond indenter into the surface of a coated compound, thus inducing massive plastic deformation to the substrate and fracture of the coating. Here, the fracture mode followed the VDI 3198 norm (German industrial standard) [28]. As shown in Figure 1, from HF1 (very good) to HF6 (very poor), each fracture mode corresponds to a varying degree of adhesion.

### 2.2. Wear Test

A S/N 17-232 pin-on-disk rotating-sliding wear tester (Swiss Center for Electronics and Microtechnology, Neuchâtel, Switzerland) was employed to evaluate the wear performance. A WC ball (WC-6 wt % Co) of 1 mm was used as the stationary pin counterpart. The coatings to be evaluated were prepared on the disks. The disks were made of SKD61 steel and with hardness about HRC 50 after heat treatment. The following conditions were used: applied normal load 5 N, rotating speed 42 rpm, rotation radius 7 mm and sliding distance 200 m. After the wear test, the surface morphologies were studied by an optical microscopy. The wear rate and depth of wear tracks were measured by scanning four cross-sections at an interval of 90 using a white-light interferometer (BMT, Nuremberg, Germany). The wear rate was calculated by the formula 2πRA/WL (10^−6^ mm^3^/N·m), where R is the rotating radius, A is an average of four measurements of a wear cross area, W is the load, and L is the sliding distance. An average of five measurements was presented.

### 2.3. Compression and Friction Test

**Figure 2:**
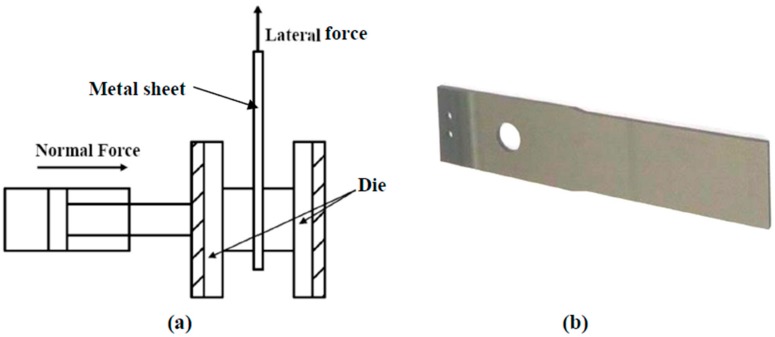
(**a**) Schematic diagram of compression and friction testing system: configuration of metal sheet, dies and applied force; (**b**) photo of metal sheet.

A compression and friction testing system was designed and assembled on a Universal Testing Machine combined with a wear tester. Figure 2a shows the configuration of a metal sheet, dies and applied forces. The metal sheet was placed between the two dies, and a specific normal force was applied to compress the sheet. A wear tester was used to provide a steady pulling force (lateral force) and to detect the resistant force. Subsequently, the detected resistant force was transformed to friction coefficient. A profilometer was used to measure the surface roughness Ra before and after tests. Here, an index Ra’/Ra was used, where Ra and Ra’ were the average surface roughness of metal sheet of three sets of measurements before and after compressing test, respectively.

A typical Mg alloy sheet is shown in Figure 2b, with a dimension 110 × 25 × 2 mm and surface roughness Ra < 0.1 µm after polishing. The JIS H4201 Mg alloy sheets were used, whose nominal composition was of: Al 3.00%, Zn 1.00%, Si 0.05%, Mn 0.2%, Ca 0.03% and Mg balance. Cubic dies of SKD61 steel were used with 25 mm in each side, and thus the contact area were 625 mm^2^. The coatings to be evaluated were prepared on the surfaces of dies in contact with the sheet. Prior to test, the contact portions were applied evenly with a graphite-base grease.

In the compressing test, the metal sheet was applied with a normal force only; that is, the sheet was kept stationary. This test aimed to evaluate the final surface roughness Ra of Mg alloy sheets after compression. The following conditions were used: normal force 12,500 N and duration 1 min.

In the friction test, the metal sheet was applied with a normal force first, and then a lateral force was applied to pull out the sheet. This test aimed to evaluate the friction coefficient during test and final surface roughness of Mg alloy sheets after friction. The following conditions were used: normal force 12,500 N, pulling velocity 10 mm/min and duration 1 min.

## 3. Results and Discussion

### 3.1. Characterization

**Figure 3:**
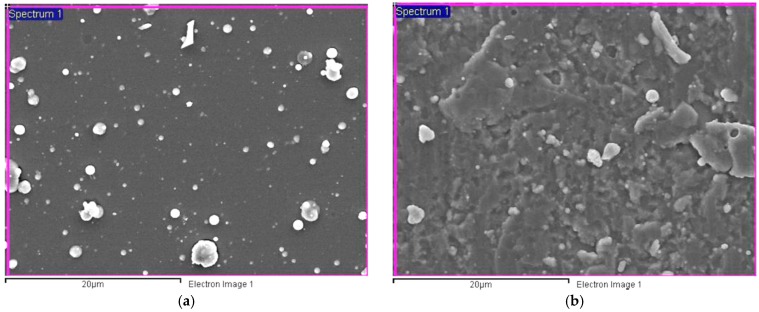
Surface morphologies of (**a**) CrTiSiN and (**b**) TiAlN coatings under SEM; Si substrate.

Figure 3 shows the surface morphologies of CrTiSiN and TiAlN coatings. The surface of CrTiSiN coating was relatively smooth than that of TiAlN coating. On both coating surfaces, micro particles were seen, which are common characteristics of coatings grown by cathodic arc deposition methods. The particles were targets materials not combined with the reactive gas. By Energy Dispersive X-ray Spectroscopy (EDX), the particles in Figure 3a were of Ti or Cr, while the ones in Figure 3b were of (Ti,Al). The surface roughness (Ra) values of CrTiSiN and TiAlN coatings were 0.25 μm and 0.33 μm, respectively.

**Figure 4:**
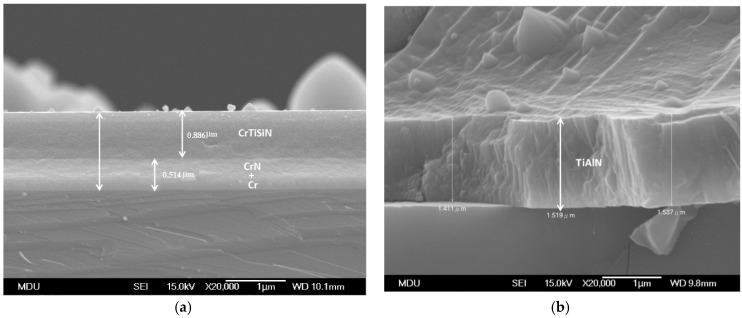
Fractured cross-sectional microstructure of (**a**) CrTiSiN and (**b**) TiAlN coatings under SEM; Si substrate.

Figure 4 shows the micrographs of cross-sectional fractured CrTiSiN and TiAlN coatings. The overall thickness of CrTiSiN coating (Figure 4a), from the interface Cr layer, the gradient CrN layer to the outmost CrTiSiN layer, was about 1.4 μm—a work by Lee and Chang [15] indicated that the addition of Si led to reduction in the width of columnar structure and then the resulting CrSiN coating was composed of CrN nanocrystallites surrounded by amorphous (a-) SiN_x_ matrix. For TiSiN coatings, the Si addition caused similar effects, resulting nanocrystallite TiN/a-SiN_x_ coating [29]. In here, the top layer was with a composition of: Ti 31.72%, Cr 22.78%, Si 2.18%, N 43.32% by EDX. The Cr and Ti elements show high solid solution to each other because the atom radii of Ti (0.145 nm) and Cr (0.125 nm) are near [16]. Therefore, it could be learned that the CrTiSiN coating was a composite consisted of nano-sized (Ti,Cr)N crystallites with a-SiN_x_ phase as matrix, showing a very featureless morphology. In comparison, the TiAlN coating showed typically columnar structure as shown in Figure 4b. It was with a thickness of about 1.5 μm including the 0.1 μm interface Ti and TiN and was with a composition of: Ti 29.82%, Al 23.00%, N 47.18% by EDX. 

**Figure 5:**
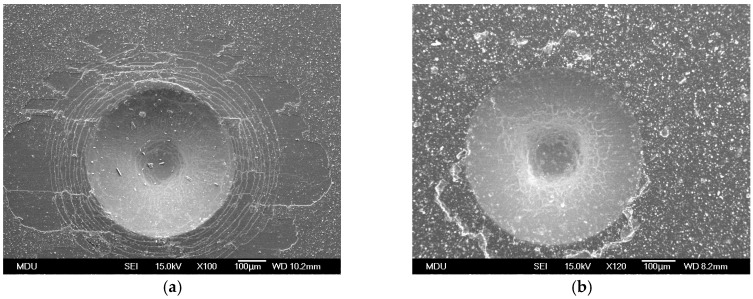
Indents on (**a**) CrTiSiN and (**b**) TiAlN coatings under SEM, using a Rockwell C hardness indentation tester.

Figure 5 shows the micro-graphs of indents on CrTiSiN and TiAlN coatings using a Rockwell C hardness indentation tester. Following the VDI 3198 norm, the CrTiSiN coating demonstrated HF3 level, while the TiAlN coating HF2. The CrTiSiN coating showed comparable adhesion to the TiAlN coating. Therefore, the CrTiSiN coated dies evaluated in the following were able to be performed without the adhesive problems.

The hardness and reduced Young’s modulus of the CrTiSiN coating were 19.35 GPa and 281.66 GPa, respectively. With the addition of Ti and Si, the CrTiSiN coating showed hardness much higher than CrSiN with 3.9% Si (8.89 GPa) [30]. For the TiAlN coating, the two values were 24.55 GPa and 412.87 GPa, respectively. Thus, the *H*^3^*/E*^2^ as well as *H/E* ratios can be calculated. The calculated *H*^3^*/E*^2^ values of CrTiSiN and TiAlN coatings were 0.0913 and 0.0868, respectively. The calculated *H/E* value of CrTiSiN and TiAlN coatings were 0.0687 and 0.0595, respectively. The hardness of CrTiSiN coating was lower than that of TiAlN coating. However, the CrTiSiN coating showed higher *H*^3^*/E*^2^ and *H/E* values than the TiAlN coating. 

### 3.2. Wear Test

**Figure 6:**
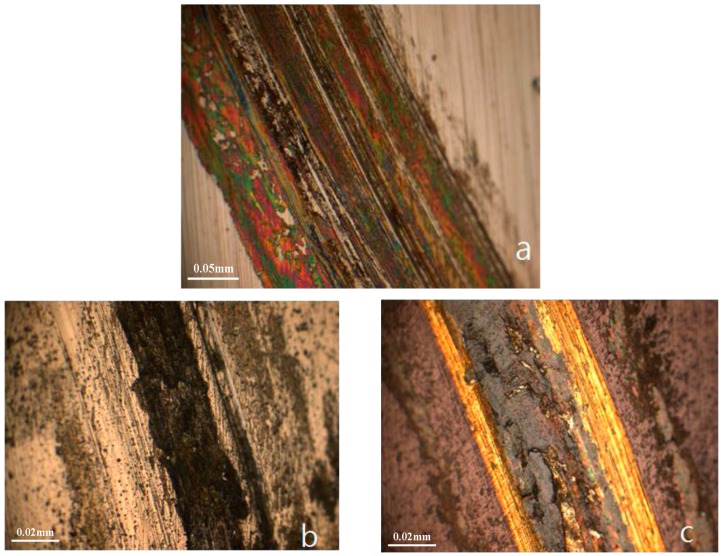
Wear tracks on: (**a**) SKD61; (**b**) CrTiSiN and (**c**) TiAlN; OM photos.

After the wear test, the uncoated SKD61 sample was severely worn, as shown in Figure 6a. The depth of wear track reached more than 1.2 µm. The wear track on CrTiSiN coating (Figure 6b) was relatively smooth. Both coatings highly improved the wear. The wear depths on CrTiSiN and TiAlN coatings were 0.482 µm and 0.692 µm, respectively. The calculated wear rate for the CrTiSiN and TiAlN coatings were 0.121 and 0.176 (10^−6^ mm^3^/N·m), respectively. Although the hardness study showed that the CrTiSiN coating (19.35 GPa) was lower than the TiAlN coating (24.55 GPa), the CrTiSiN coating indicated better wear resistance than the TiAlN coating. As widely accepted criteria, the *H*^3^*/E_r_*^2^ rule allows to estimate the material’s ability to dissipate energy at plastic deformation during loading [31], while the *H/E* rule characterizes the resistance of the material to elastic deformation [32]. Experience considered that the ranking of materials according to their *H/E* ratio can provide extremely close agreement to their ranking in terms of wear [33]. In here, the *H*^3^*/E_r_*^2^ and *H/E* rules can be applied well. The CrTiSiN coating possessed a higher *H*^3^*/E_r_*^2^ and *H/E* values than the TiAlN coating. 

**Figure 7:**
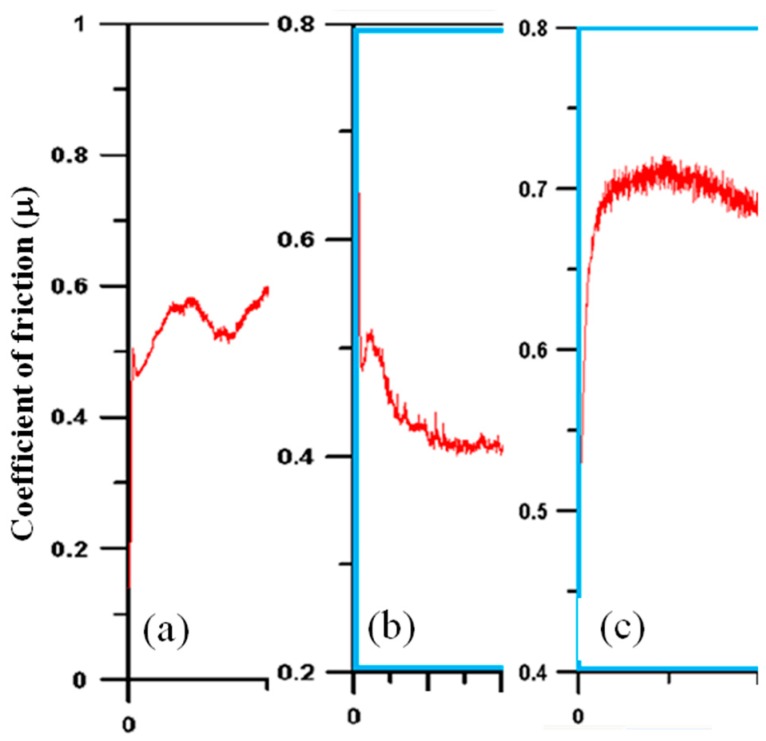
Evolution of friction coefficient (μ) against sliding distance: (**a**) uncoated SKD61; (**b**) CrTiSiN coating; and (**c**) TiAlN coating.

Figure 7 shows the evolution of friction coefficients for the three samples against sliding distance. In Figure 7a, the uncoated sample showed that the friction coefficient kept increasing slowly as sliding continued until reaching 0.6. For the CrTiSiN coating (Figure 7b), the friction coefficient dropped gradually after running-in and then reached a steady value 0.40. Comparatively, the TiAlN coating (Figure 7c) showed friction coefficient increased fast after running-in, and then reached a steady value 0.70, which was higher than the uncoated sample. 

### 3.3. Compression and Friction Test

**Figure 8:**
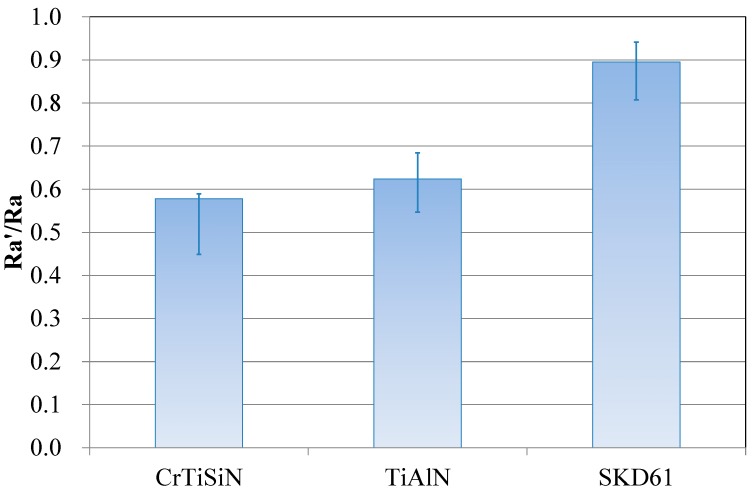
Ra’/Ra values of metal sheet surface after compression test; Ra and Ra’ being average surface roughness before and after test.

The results of compressing test is shown in Figure 8. Both the coatings provided an improvement by 25–30% in surface roughness of Mg alloy sheets. The Mg alloy sheet in the CrTiSiN pair showed better surface condition than that in the TiAlN pair. 

**Figure 9:**
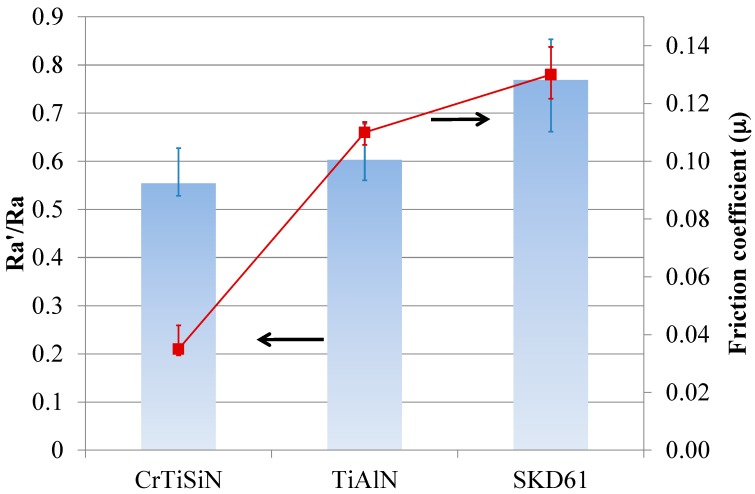
Ra’/Ra values of metal sheet surfaces after friction test and steady fiction coefficient values during friction test; Ra and Ra’ being average surface roughness before and after test.

Figure 9 shows the results of friction test. The CrTiSiN pair showed much lower friction coefficient than the TiAlN pair under high compression pressure. Meanwhile, the Mg alloy sheet in the CrTiSiN pair showed a lower Ra’/Ra value than that in the TiAlN pair.

From Figure 8 and Figure 9, it is learned that good surface condition of compressed Mg alloy sheets and low friction coefficient were obtained by using the CrTiSiN coating on the compressing dies. Especially, the ultra-low friction coefficient of the CrTiSiN pair reaching 0.035 was very attractive as compared with the uncoated pair 0.13. The surface roughness condition of parts after high pressure compressing operation is largely influenced by the surface roughness condition of contact face of compressing tools. That is, to have a good condition surface (low surface roughness) of compressing tools is a direct way to achieve good surface of compressed parts. Meanwhile, a good condition surface between the compressing tool and compressed part helps to reduce the friction when relative movement occurs. In this study, the SKD61 die was with lowest initial Ra value (<0.1 µm). After compression test and friction test, the Mg alloy sheet showed the highest Ra’/Ra value. Comparatively, the Mg alloy sheet showed a significantly low Ra’/Ra value and friction coefficient when the CrTiSiN coated die was used despite of higher initial Ra value (0.25 μm). This resulted from the high deformation resistance of CrTiSiN coating that provided a good condition surface in contact with the compressed part under high compressing pressure. The friction coefficient was also decreased at the same time.

On the basis of above result, when forming Mg alloy sheets the CrTiSiN coating is a good choice for dies or molds to achieve better condition surface and lower friction.

## 4. Conclusions

The number of products made of Mg alloys is increasing. However, forming of Mg alloys still presents some problems. This study presented our research on preparing a composite CrTiSiN coating and designing and assembling of a self-designed high pressure compression and friction tester. The composite CrTiSiN coating, with a single layer TiAlN coating as reference, was then characterized and studied for wear resistance and high pressure compression and friction behavior. The JIS H4201 Mg alloy sheets were used as the tested material in the compression and friction tester. We obtained the following conclusion: the hardness of CrTiSiN coating was lower than that of TiAlN coating. However, the CrTiSiN coating showed better wear resistance than the TiAlN coating. In view of wear performance, both the *H/E* and *H*^3^*/E_r_*^2^ rules can be applied well here for CrTiSiN coating showing the higher values. In the compressing test, a better surface condition of Mg alloy sheet was obtained by CrTiSiN coated dies than by TiAlN coated ones under high compression pressure. In the friction test, the CrTiSiN coating pair that showed ultra-low friction reaching 0.035 was very attractive. It had a value of only 25% of the uncoated pair.
